# More than Beauty—Edible Flowers of New *Rosa* × *hybrida* Genotypes Grown in Vojvodina (Serbia) as a Novel Source of Bioactive Compounds

**DOI:** 10.3390/plants15203099

**Published:** 2026-10-10

**Authors:** Nemanja Živanovic, Marija Lesjak, Dejan Orčić, Biljana Božanic Tanjga, Mirjana Ljubojević, Jelena Čukanović, Nataša Simin

**Affiliations:** 1 Department of Chemistry, Biochemistry and Environmental Protection, Faculty of Sciences, University of Novi Sad, Trg Dositeja Obradovića 3, 21000 Novi Sad, Serbia; nemanja.zivanovic@dh.uns.ac.rs (N.Ž.); marija.lesjak@dh.uns.ac.rs (M.L.); dejan.orcic@dh.uns.ac.rs (D.O.); 2 Breeding Company ‘Pheno Geno Roses’, Maršala Tita 75, 23326 Ostojićevo, Serbia; b.bozanic@phenogenoroses.com; 3 Department of Fruit Growing, Viticulture, Horticulture and Landscape Architecture, Faculty of Agriculture, University of Novi Sad, Trg Dositeja Obradovića 8, 21000 Novi Sad, Serbia; mirjana.ljubojevic@polj.uns.ac.rs (M.L.); jelena.cukanovic@polj.uns.ac.rs (J.Č.)

**Keywords:** rose petals, edible flowers, polyphenols, antioxidants, acetylcholinesterase, α-amylase, α-glucosidase

## Abstract

Flowers have a long tradition of use beyond ornamental purposes, including incorporation into numerous food products. Roses stand out for their attractive colors and pleasant floral aromas, yet their nutraceutical potential remains largely underestimated. In this study, rose flowers from five new garden cultivars were evaluated for their potential use as food ingredients. Total phenolic, total flavonoid, total monomeric anthocyanin and vitamin C contents were quantified, along with quantitative analysis of 45 selected compounds by LC-MS-MS. Additionally, in vitro antioxidant, neuroprotective and hypoglycemic activities were assessed. They proved to be rich sources of polyphenols (TPC in the range of 122–240 mg GAE/g de, TFC in the range of 18.1–42.7 mg QE/g de) and vitamin C (0.24–0.31 mg/g fw), exhibiting strong antioxidant and moderate neuroprotective effects. They are weak inhibitors of α-amylase and strong inhibitors of α-glucosidase (23.7–81.6% inhibition at 2.50 μg/mL), suggesting potential benefits for managing blood sugar levels and metabolic health. Cultivar ‘Magic Aroma’ demonstrated the greatest potential for further research and incorporation into food products. Given modern consumers’ growing interest in natural and functional food ingredients, these findings highlight the untapped potential of rose flowers for innovative, health-promoting culinary uses.

## 1. Introduction

The global edible flowers market was valued at USD 0.421 billion in 2023 and is projected to reach USD 0.66 billion by 2032, growing at a compound annual growth rate (CAGR) of 5.20% between 2024 and 2032. This growth is driven by increasing consumer interest in natural ingredients, evolving dietary habits and the incorporation of edible flowers in the food, beverage and nutraceutical sectors [1].

In addition to nutritional components such as proteins, carbohydrates, lipids, free amino acids, and organic acids, flowers also contain a diverse range of bioactive compounds—including carotenoids, polyphenols, and terpenoids—whose distribution varies among floral parts such as petals, nectar, and pollen. Petals, in particular, have been identified as a rich source of vitamins, minerals and polyphenols. Although polyphenols such as flavonoids do not contribute directly to nutritional value, their numerous beneficial effects on human health, such as the prevention of neurodegenerative and cardiovascular diseases, underscore the potential of flowers as functional foods [2,3,4,5]. However, despite their growing culinary and commercial appeal, scientific research on edible flowers remains limited, particularly in terms of their compositional analysis, safety evaluation and functional applications.

Known as the “queen of flowers”, the rose has had great value throughout history, not just as an ornamental plant but also in medicine, religion and the arts. Roses have a long tradition of use as food, such as jams, desserts and beverages, and are increasingly gaining prominence in traditional and luxury cuisines worldwide. Today, the genus *Rosa* encompasses over 200 species and over 37,000 cultivars, but this number is probably even higher due to the constant development of new genotypes. Like several other edible flowers, rose petals are a rich source of sugars and carbohydrates, comprising 60–86% of their dry weight, levels comparable to those found in bananas. Additionally, they contain significant amounts of crude proteins (2–12% dry weight) and are low in fats with less than 4% dry weight [6,7]. Moreover, they are a rich source of polyphenols (phenolic acids, flavonoids and anthocyanins) [8,9], vitamins (C, B1, B2, B3, B5, B6 and B9) [9,10] and minerals (K, P, Ca, S, Mg, Na, Fe, Cu, Zn) [11,12]. Rose flowers exhibit proven in vitro anti-inflammatory, neuroprotective, anti-diabetic, antimicrobial, antifungal, anti-aging, antiviral and antioxidant activities, which are mostly attributed to high levels of polyphenols [6,8,9,10].

Furthermore, the most recent models developed for choosing edible flowers rely on assigning appropriate weights to different evaluation criteria, as determined by expert judgment [13]. According to the conducted analysis, experts ranked sensory qualities (0.175), antioxidant potential (0.163) and nutritional composition (0.146) as the most influential factors in the selection process, positioning the organoleptic properties before the nutritional ones. Organoleptic properties, such as taste, aroma and texture, are particularly important in shaping their appeal, since consumers are not always accustomed to the nutrient content, but to what they see and sense. Edible roses exemplify this trend, as their characteristic fragrance, delicate flavor and high content of bioactive compounds make them one of the most prominent and widely appreciated edible flowers in gastronomy and functional foods [14].

Over the past decade, the company Pheno Geno Roses has developed a diverse range of rose cultivars through targeted hybridization and controlled pollination, aiming to produce offspring with desirable traits such as disease resistance, increased flower number and size, varied colors, and distinctive fragrances and aromas [15]. Through standardized sensory analysis, five genotypes emerged as particularly promising. In this study, petals from these newly developed rose genotypes, cultivated in Serbia, were analyzed for the first time to enhance their commercial appeal and explore their potential applications in the food, cosmetic and pharmaceutical industries. All investigated genotypes belong to *Rosa* × *hybrida* and were produced exclusively through conventional breeding methods without chemical protection.

Importantly, the chemical composition and biological activities of rose petals from novel genotypes remain largely unexplored. By chemically characterizing petal extracts, assessing total phenolic, flavonoid, monomeric anthocyanin and vitamin C contents, as well as specific polyphenolic compounds and evaluating biological activities such as in vitro antioxidant, neuroprotective and hypoglycemic effects, this study will contribute to the estimation of their potential to be used as functional foods.

## 2. Results and Discussion

### 2.1. Assessment of Morphological Characteristics Relevant to Feedstock Yield and Cultivar Selection

Key plant traits, such as shrub size, and the number of shoots and laterals, are important criteria for cultivar selection when considering roses as a source of edible flowers and raw material for processing [9]. Out of five investigated genotypes, only one, ‘Gentle Aroma’ (GA), is characterized by the formation of flowering laterals (2.6 in average per shrub, and 3.2 flowers per lateral), while the remaining four form only the dominant flowering shoot with one flower at the top (Table 1). Flowers of the investigated cultivars are double, pale-colored, fragrant (scores 4–5), abundant in petals (52–148.4) and with diameters from 6.72 cm to 8.04 cm. Cultivar ‘Magic Aroma’ (MA) achieved the highest values for both petal length (PL) and petal width (PW), with 4.8 cm for PL and 4.18 cm for PW (Table 1). The formation of flowering laterals in GA may contribute to a higher flower yield, while the large petals of MA may provide a greater amount of usable petal material per flower.

### 2.2. Sensory Evaluation

Due to the high scores for fragrance (4–5), subsequent sensory evaluation was performed. Results revealed that in the top notes (Table 2), only a few were distinguished with more or closely around the 20 scoring percentages (lemon note in ‘Intense Aroma’ (IA) and GA, camphor in ‘Unique Aroma’ (UA) and anise in ‘Natural Aroma’ (NA)). Middle notes, divided into 21 different notes, exhibited lower scoring percentages, with several reaching more than 10% (flowery in IA, GA and UA, forest in IA, fruity in IA and UA, sweet honey in GA, NA and MA, as well as spicy in NA). Due to fewer described base notes, percentages for these properties were again higher as they were for top notes. Namely, more than 15% scoring for earthy, balsamic and musky notes was obtained in MA, sandalwood, musky and vanilla in GA, as well as vanilla in IA.

Considered altogether, the present notes evoked different emotions in panelists (Table 3). The most pronounced emotion was intensive by MA, followed by mild in GA as well as heavy and intensive by NA. Interestingly, NA and MA were highly scored for irritating emotion. While scores were generally scattered and diverse, association with rich smell was uniform for all five investigated varieties, taking values from 6.7% for UA to 9.5% for GA. All five cultivars were also more uniformly scored as inspiring, with percentages between 5.9 and 10.3%.

Consistent with previous findings that food-induced emotions strongly influence food choice and can shape craving, calmness, or irritability [16], our results suggest that the distinct emotional responses evoked by edible rose cultivars may play a decisive role in their acceptance and culinary application. According to Najar et al., like their decorative value, flowers’ aromas can provoke varied emotions, reflecting each individual’s unique memories and experiences [17]. In line with this, cultivars like GA with honey, vanilla, and sandalwood notes may promote soothing impressions, while the more intense, spicy-fruity profiles of NA and MA may explain their stronger association with irritation among panelists.

### 2.3. Chemical Characterization

Chemical characterization of the investigated rose petals was achieved by determining total phenolic, flavonoid, monomeric anthocyanin and vitamin C contents (Table 4) and by quantitative analysis of selected phenolic compounds by LC-MS/MS (Table 5).

Results showed that in the examined samples, total phenolic content (TPC) was in the range of 122–240 mg GAE/g de (10.9–23.4 mg GAE/g fw), with the highest amount detected in petals of genotype MA, followed by NA (180 mg GAE/g de) (Table 4). These results are in accordance with previously examined rose cultivars in our laboratory. Namely, the TPC of previously examined genotypes was between 148–260 mg GAE/g de [8] and 91–271 mg GAE/g de [9]. Furthermore, Kumari et al. investigated 50 varieties of *R. hybrida* grown in India [18]. They reported TPC in petals to be in the range of 0.05–42.8 mg GAE/g fw, with the majority of cultivars containing less than 10 mg GAE/g fw. Mlcek et al. reported a similar result, with 4.45 mg GAE/g fw for roses grown in Czechia [19]. Correspondingly, compared with the results of others, the cultivars analyzed in this study represent a rich source of phenolic compounds, particularly cultivar MA. When comparing the total phenolic content (TPC) of the rose cultivars investigated in this study with that of other edible flowers, such as *Tagetes* spp. (marigold; 212.9 mg GAE/g de), *Cosmos* spp. (sulfur cosmos; 102.5 mg GAE/g de), *Antigonon* spp. (coral vine; 177.2 mg GAE/g de) and *Bougainvillea* spp. (paper flower; 138.2 mg GAE/g de) [20], as well as *Rosa* spp. (damask rose; 72.6 mg GAE/g de), *Viola* spp. (wild pansy; 48.6 mg GAE/g de), *Hibiscus* spp. (Chinese hibiscus; 28.2 mg GAE/g de) and *Sambucus* spp. (elderflower; 30.9 mg GAE/g de) [21], it becomes evident that the edible petals of the newly assessed rose genotypes possess comparable or even higher levels of polyphenols. These results highlight their strong potential as valuable dietary sources of bioactive phenolic compounds.

Total flavonoid content (TFC) of the rose genotypes examined in this study ranged from 18.1 to 42.7 mg QE/g de, with the highest levels observed in the genotypes NA, GA and MA (Table 4). These results are consistent with our previous findings for other rose genotypes grown in Serbia, where TFC values ranged from 17.3 to 59.8 mg QE/g de [8,9]. Mlcek et al. reported 2.43 mg RE/g fw in rose flower extract grown in Czechia [19], which is comparable with the extracts in this study. TFC of other edible flowers, such as *Viola* spp. (wild pansy; 25.9 mg QE/g de), *Rosa* spp. (rose; 9.1 mg QE/g de), *Sambucus* spp. (elderflower; 9.4 mg QE/g de), *Hibiscus* spp. (Chinese hibiscus; 2.6 mg QE/g de) [21], *Anchusa* spp. (large blue alkanet; 17.3 mg QE/g de), *Hedysarum* spp. (French honeysuckle; 38.6 mg QE/g de), *Malva* spp. (common mallow; 12.7 mg QE/g de), *Capparis* spp. (caper bush; 19.4 mg QE/g de) and *Robinia* spp. (black locust; 45.3 mg QE/g de) [22], highlights that edible flowers, including those analyzed in the present study, can be valuable sources of dietary flavonoids.

Total anthocyanin content (TAC) in UA and MA samples, which have pink-colored petals, was 270 and 1749 µg CE/g DE, respectively (Table 4). In contrast, the TAC in the IA, NA and GA rose cultivars, characterized by yellow pigmentation, was below the quantification limit of the applied analytical method (Table 4). These results were expected, since anthocyanins are the pigments primarily responsible for pink-to-red flower coloration. These findings are consistent with previous studies demonstrating a positive correlation between vivid flower coloration and elevated anthocyanin content [8,9,18,23].

Plants and most animals are able to synthesize vitamin C (ascorbic acid), but humans and some other mammals have lost that ability and need to obtain it through food. Namely, humans lack the functional gene for the enzyme gulonolactone oxidase, a terminal enzyme in ascorbic acid synthesis. Vitamin C is not only a powerful natural antioxidant, but also serves as a cofactor for many enzymes such as prolyl 4-hydroxylase (necessary for collagen synthesis and the reason why vitamin C deficiency causes scurvy) and dopamine β-monooxygenase (involved in norepinephrine biosynthesis). The recommended dietary allowance is 90 mg for men and 75 mg for women [24]. Rose genotypes investigated in this study contained 236–309 µg vitamin C/g fw, with the highest amount detected in MA (Table 4). These results are similar to previously reported values for edible cultivars of *R. hybrida* investigated by our group, with vitamin C content between 111 and 479 µg/g fw [9]. These results show that rose flowers are a good source of vitamin C and are comparable with other fruits and vegetables known as good sources of dietary vitamin C, such as strawberry (100–800 µg/g fw), melon (100–290 µg/g fw), lemon (230 µg/g fw), bitter orange (440 µg/g fw), grapefruit (356 µg/g fw) and orange (412 µg/g fw) [25,26]. Compared to other edible flowers, such as *Taraxacum* spp. (dandelion; 341 µg/g fw), *Viola* spp. (sweet violet; 1227 µg/g fw), *Trifolium* spp. (white clover; 428 µg/g fw), *Begonia* spp. (tuberous begonia; 69.9 µg/g fw), *Iris* spp. (leopard lily; 2028 µg/g fw), *Rosa* spp. (rose; 599 µg/g fw) and *Tagetes* spp. (Mexican marigold; 180 µg/g fw), it can be concluded that roses investigated herein are a moderate source of vitamin C, which enhances their nutritional value [27].

Quantitative analysis of selected polyphenols (Table 5) revealed that methanol extracts of rose petals contained significant amounts of quinic acid (9.4–16.3 mg/g de), rutin (0.25–1.5 mg/g de), kaempferol 3-*O*-glucoside (5.7–40.6 mg/g de), quercitrin (0.5–7.7 mg/g de) and quercetin 3-*O*-glucoside+galactoside (1.6–11 mg/g de) (Table 5). These results are in accordance with our previous results for other rose genotypes grown in Serbia [8,9] and with results obtained by Mikanagi et al. [28]. In a study on rats, quercetin and quinic acid showed hypoglycemic activity in diabetic rats with a synergistic effect when used together. They increased insulin levels, improved lipid status and showed a protective effect on kidneys in diabetes [29]. Quercetin 3-*O*-glucoside is another compound with anti-diabetic activity; additionally, it also provides a protective effect on the kidneys [30]. Moreover, quercetin 3-*O*-glucoside supplementation was shown to increase bone formation [31]. Quercetin 3-*O*-galactoside, hyperoside, has numerous beneficial effects on health, such as neuroprotective, anti-cancer, renal-protective, antioxidant, anti-diabetic and others [32]. Kaempferol possesses antioxidant, antimicrobial, anti-cancer, anti-inflammatory and other activities [33]. Although achieving the same doses of individual compounds used in the aforementioned studies would require the intake of several grams of rose petal extracts, this does not imply that using smaller amounts of extracts or fresh petals in the everyday diet would not be beneficial, especially considering the potential synergistic effects of different compounds in the extract.

**Figure 1:**
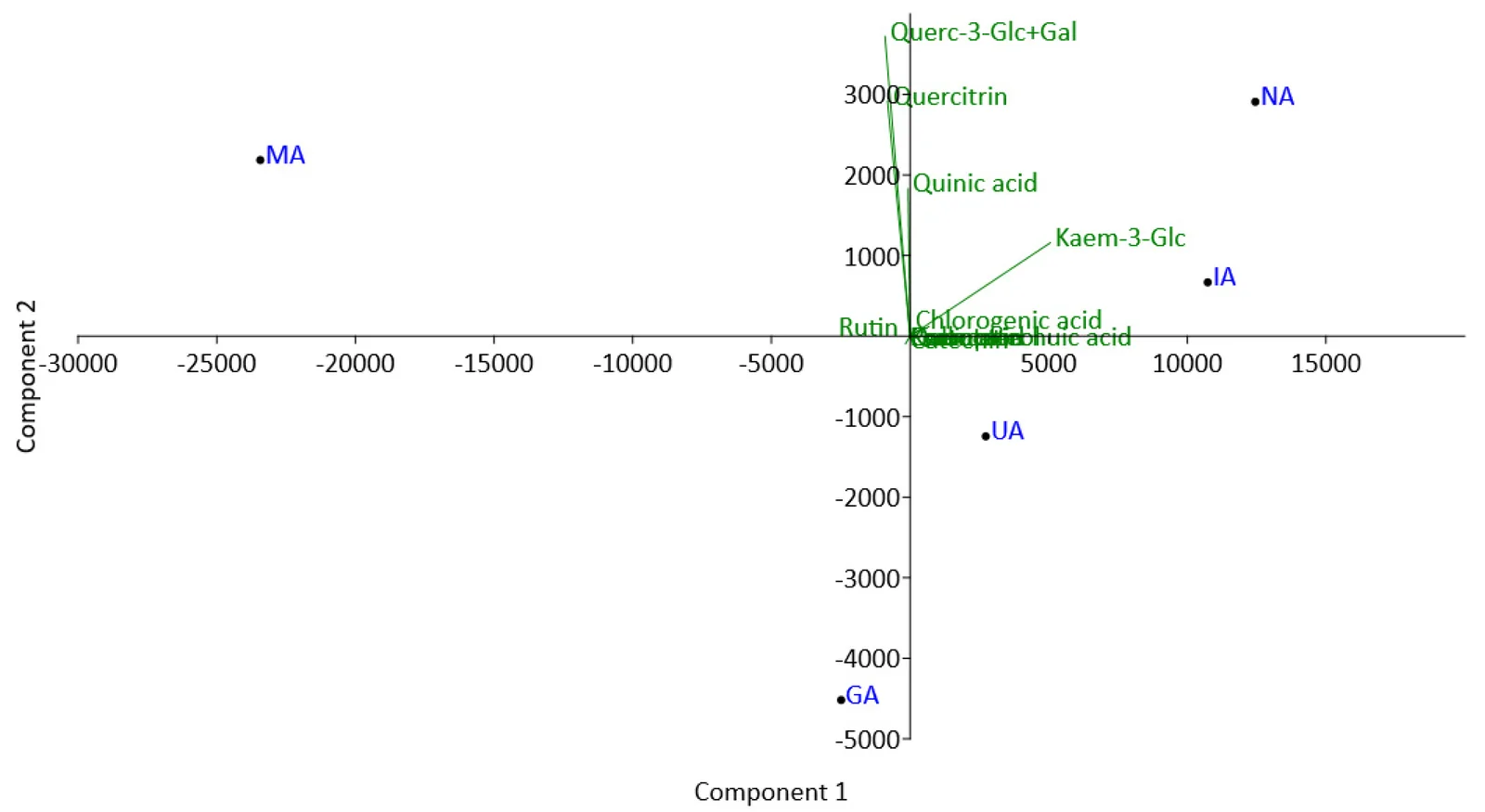
PCA biplot (PC1 vs. PC2) of the 12 dominant polyphenolic compounds in methanolic extracts of rose petals.

Principal component analysis (PCA) of the 12 most abundant polyphenolic compounds in the methanol extracts of rose petals illustrated compositional variation among the examined edible rose genotypes, as shown in Figure 1. Principal Component 1 (PC1) accounted for 92.91% of the total variance, while Principal Component 2 (PC2) explained an additional 3.98%. Compared to other genotypes, NA and IA are characterized by a high content of quinic acid and kaempferol 3-*O*-glucoside and moderate amounts of quercitrin and quercetin 3-*O*-glucoside+galactoside, and are located in the upper right quadrant of the biplot. Genotype MA is characterized by a high content of quinic acid, like NA and IA, but unlike them, MA is also rich in quercitrin and quercetin 3-*O*-glucoside+galactoside and has a low content of kaempferol 3-*O*-glucoside, placing it in the upper-left quadrant of the biplot. UA is characterized by a high content of kaempferol 3-*O*-glucoside and moderate amounts of quercitrin, quercetin 3-*O*-glucoside+galactoside, and rutin, and the lowest amount of quinic acid compared to other genotypes, placing it in the lower-right quadrant near the center of the biplot. Finally, genotype GA is also located in the lower-left quadrant, near the boundary with the right quadrant. It is characterized by a high content of quinic acid, kaempferol 3-*O*-glucoside, and rutin and low amounts of quercitrin and quercetin 3-*O*-glucoside+galactoside compared to other investigated genotypes.

**Figure 2:**
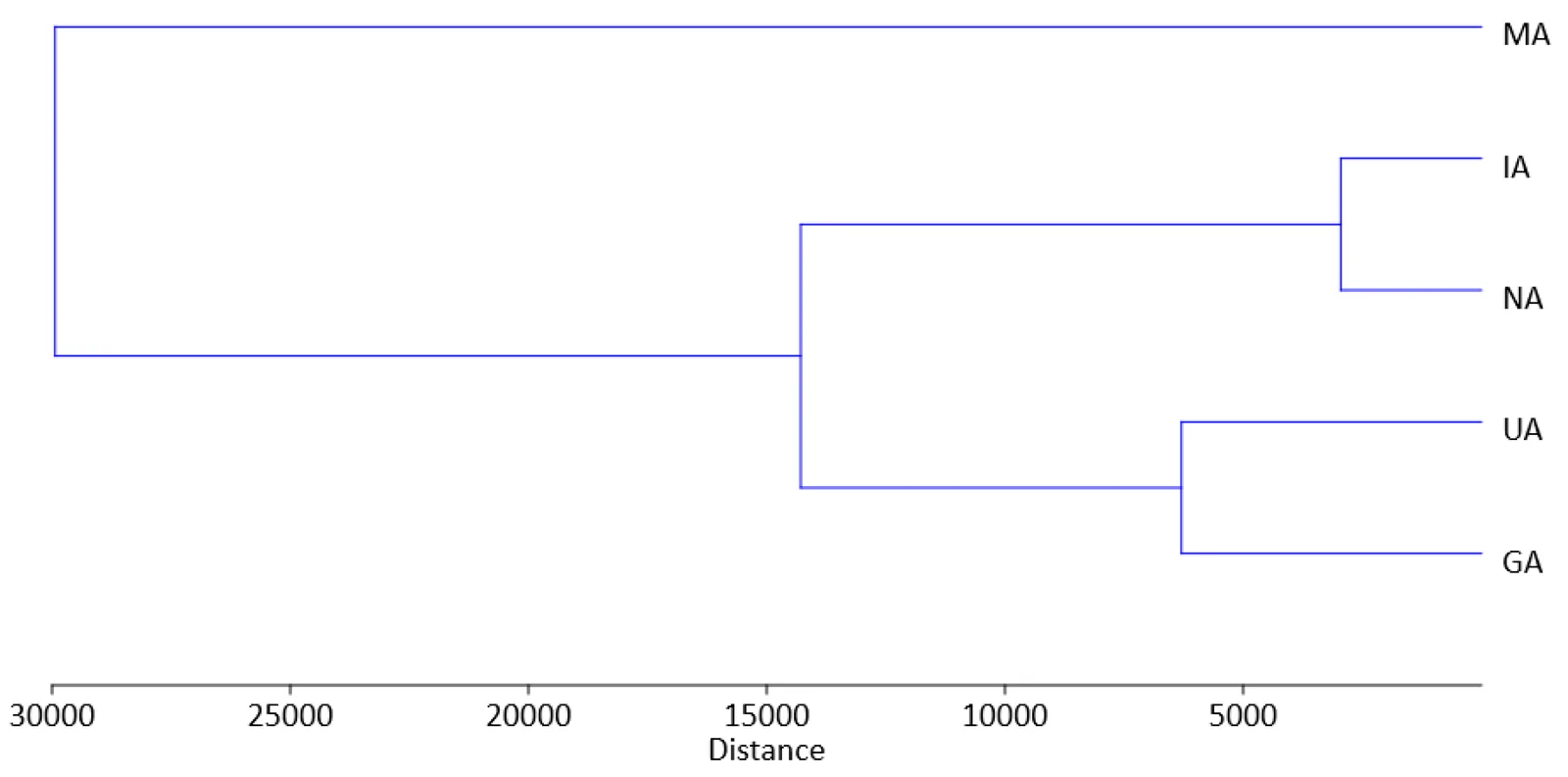
Dendrogram obtained by hierarchical clustering analysis of 12 most dominant compounds determined in rose petal methanol extracts.

A dendrogram generated by hierarchical clustering analysis of the 12 most dominant polyphenolic compounds identified in the methanolic extracts of the investigated rose petals is presented in Figure 2. It was obtained using Ward’s method, and the distance represents the differences in chemical composition. Similarly to the biplot obtained for PCA, MA is separated from the other four genotypes, which are further divided into two subgroups, one consisting of IA and NA and the other with genotypes UA and GA.

### 2.4. In Vitro Evaluation of Biological Activities

The antioxidant (DPPH and FRAP assays), neuroprotective (acetylcholinesterase inhibition assay), and hypoglycemic (α-amylase and α-glucosidase inhibition assays) activities of edible rose petal extracts were evaluated and the results are presented in Table 6.

Oxidative stress is associated with numerous diseases, including atherosclerosis, ischemia/reperfusion injury, inflammation, Alzheimer’s disease and cancer. One potential strategy for the prevention and treatment of these pathological conditions is the intake of antioxidants—compounds capable of mitigating oxidative stress through multiple mechanisms, including free radical scavenging, chelation of transition metals such as iron, inhibition of lipid peroxidation, and activation of endogenous antioxidant defense systems via upregulation of antioxidant enzymes (glutathione peroxidase, superoxide dismutase, and catalase) or activation of the Nrf2 signaling pathway. The reduction of oxidative stress through multiple mechanisms underlies their other biological effects, including anti-inflammatory, neuroprotective, cardioprotective, and anti-cancer actions [34,35,36]. Although DPPH and FRAP are relatively simple in vitro assays, they are widely used for the preliminary evaluation of antioxidant activity and offer useful information on the antioxidant potential of plant extracts and compounds. Methanol extracts of rose petals from genotypes examined in this study showed great antioxidant activity with EC_50_ ranging from 18.4 to 44.9 µg/mL in the DPPH assay (Table 6). The highest activity was exhibited by genotype MA, followed by NA and GA. Similar results were obtained in our previous studies of *R. hybrida* genotypes with EC_50_ values ranging from 9.24–42.5 µg/mL [9] to 17.7–27.8 µg/mL [8]. Abdel-Hameed et al. reported an EC_50_ of 49.4 µg/mL for the methanol extract of defatted Taiff rose petals [37]. Compared to other edible flowers, roses express similar DPPH scavenging activity to *Dahlia* spp. (dahlia; EC_50_ = 0.63 µg/mL), *Centaurea* spp. (cornflower; EC_50_ = 0.83 µg/mL) [38] and *Sophora* spp. (shrubby sophora; EC_50_ = 20.7 µg/mL) [39], and much higher activity than *Calendula* spp. (pot marigold; EC_50_ = 1.37–15.97 mg/mL) [38] and *Helianthus* spp. (sunflower; EC_50_ = 5.99–13.09 mg/mL) [40]. In the FRAP assay, the examined extracts exhibited strong antioxidant activity with values between 62.6 and 173 mg AAE/g de. MA was the most active, followed by GA and NA. These results followed the same trend as results from the DPPH assay. Previous results regarding the FRAP assay showed similar values for other *R. hybrida* petals, ranging between 74.6–227 mg AAE/g de [9] and 132–209 mg AAE/g de [8]. Compared to edible flowers from 48 peony cultivars, which exhibited FRAP antioxidant activity ranging from 4.80 to 17.9 mg AAE/g fw, the rose cultivars analyzed in this study demonstrated similar levels of activity (5.47–16.9 mg AAE/g fw) [41]. Due to their high content of natural antioxidants, such as polyphenols and vitamin C, and demonstrated antioxidant activity, the edible rose flowers examined in this study have potential as functional ingredients in food products. Sik et al. reported that incorporating edible flowers, particularly roses, into biscuits significantly enhanced their antioxidant capacity [42]. Notably, the addition of rose petals had no negative impact on consumer acceptance, which the authors attributed to the pleasant floral and sweet aroma of the rose. Similarly, Kumar et al. developed a functional beverage using finger millet, oats, and double-toned milk, and found that the addition of rose syrup not only increased the total antioxidant capacity of the beverages but also improved their sensory attributes, enhancing consumer appeal [43].

Acetylcholine is a signal transmitter in synapses of central and peripheral neurons and is involved in cholinergic response. Acetylcholinesterase (AChE) is an enzyme responsible for the degradation of acetylcholine in synapses and signal termination. Inhibition of this enzyme has been one of the treatments for neurological diseases such as Alzheimer’s, although it can only treat symptoms. Successfully applied inhibitors include the alkaloids galantamine and physostigmine (eserine), but their use is associated with adverse effects such as hepatotoxicity. In search of new possible therapeutics, flavonoids have shown some promising results [44].

The new rose genotypes examined in this study exhibited the ability to inhibit AChE, with values ranging from 6.3 to 12.9 ng EE/g de, corresponding to 20.9–33.3% inhibition at a concentration of 50 µg/mL. The highest activity was exhibited by MA, IA and NA. These results are in accordance with previously reported data for other *R. hybrida* petal extracts, with values ranging from 16.3 to 68.5 ng EE/g de [9], and from 10.2 to 18.3 ng EE/g de [8]. Extracts of flowers from *R. damascene* cultivars showed slightly higher anti-AChE activity, with IC_50_ values ranging from 22.9 to 32.03 µg/mL, while cultivar Comlex-635 exhibited much stronger inhibitory potential with IC_50_ of 3.92 µg/mL [45]. In the study by Esmaeli et al., *R. damascena* flower and *R. canina* fruit extract achieved 10.9 and 31.3%, respectively, at a concentration of 300 µg/mL [46], indicating lower inhibitory potential compared to the *R. hybrida* genotypes examined in this study. In contrast, extracts from edible flowers of *Nymphaea* spp. (water lilies), which are commonly consumed, demonstrated similar inhibitory potential ranging from 38.9% to 63% at 47.17 µg/mL [47]. On the other hand, the rose flowers investigated in this study exhibited significantly higher inhibitory potential than flowers of yellow and orange cultivars of African marigold (*Tagetes* spp.), which achieved 50% inhibition at concentrations of 1.13 and 1.25 mg/mL, respectively [48]. Moreover, Yamamoto and Kono reported an IC_50_ of 60.9 µg/mL for *Rosa miniature* extract [49]. Although the rose petal extracts investigated herein are not highly potent AChE inhibitors, they exhibit measurable inhibitory activity that may be beneficial for the prevention, rather than treatment, of neurodegenerative diseases. This potential is further supported by their richness in flavonoids, which have demonstrated neuroprotective effects through various mechanisms, including the prevention of oxidative neuronal damage and inhibition of neuron death via ferroptosis [36].

Diabetes mellitus is a metabolic disorder characterized by high blood glucose levels and disrupted metabolism of carbohydrates, proteins and lipids caused by impairment or destruction of insulin-secreting cells (pancreatic β-cells). Diabetes mellitus is classified into several types: type 1 (insulin-dependent), type 2 (insulin-independent) and type 1.5, which is considered an autoimmune disorder. Hyperglycemia associated with diabetes is linked to serious damage to multiple organs, including the heart, nerves and kidneys. One effective strategy to reduce postprandial hyperglycemia involves the inhibition of enzymes responsible for carbohydrate hydrolysis [50].

In this study, rose petal extracts were tested for their ability to inhibit α-amylase and α-glucosidase. Extracts showed low potential for inhibition of α-amylase with results in the range of 30.8 to 57.9 mg ACAE/g de or 3.75 to 6.97% at a concentration of 277 µg/mL. The most active genotypes were NA and MA, followed by IA and UA, while GA showed no inhibitory activity toward α-amylase. These results are in accordance with previous results for other *R. hybrida* flowers with results in the range of 7.3 to 95.6 mg ACAE/g de [8]. On the other hand, rose flower extracts exhibited strong inhibitory activity toward α-glucosidase, with results in the range of 12.2 to 212 g ACAE/g de or 23.7 to 81.6% at a concentration of 2.50 µg/mL. The highest activity was exhibited by genotype MA, followed by NA and GA. These results are consistent with previously reported data for rose petals, which showed values ranging from 38.6 to 339 g ACAE/g de [8]. This suggests that rose extract may not inhibit the hydrolysis of carbohydrates such as starch by oral α-amylase into smaller fragments, but rather inhibits the hydrolysis of these fragments by intestinal α-glucosidase, thereby reducing glucose release from carbohydrates and subsequent absorption and transport into the bloodstream. This high α-glucosidase inhibition potential can be, at least to some degree, attributed to the presence of flavonoids such as quercetin and kaempferol glycosides, which are present in rose petal extracts in high amounts [50,51]. In contrast to the rose extracts, marigold cultivars (*Tagetes* spp.) exhibited IC_50_ values in the ranges 2.38–4.87 mg/mL and 3.12–7.40 mg/mL toward α-amylase and α-glucosidase, respectively [52]. IC_50_ values for α-amylase inhibition reported by Yamamoto and Kono include *Rosa* spp. (miniature rose; 232 µg/mL), *Dendrobium* spp. (phalaneopsis orchid; 162 µg/mL), *Viola* × *wittrockiana* cv. *fluna* (268 µg/mL) and *Viola* × *wittrockiana* cv. *pansy* (183 µg/mL). Regarding α-glucosidase inhibition, IC_50_ values were found to be 0.41 µg/mL for *Chrysanthemum morifolium*, 80.3 µg/mL and 133.9 µg/mL for *Viola* × *wittrockiana* cvs. *fluna* and *pansy*, respectively, and 79.4 µg/mL for *Primula* × *Juliana* [49]. These findings further underscore the hypoglycemic potential of edible flowers, particularly the roses examined in this study, which demonstrated strong inhibitory activity against α-glucosidase. This inhibitory potential warrants further investigation to explore their possible applications in managing postprandial hyperglycemia.

## 3. Materials and Methods

### 3.1. Plant Material

**Figure 3:**
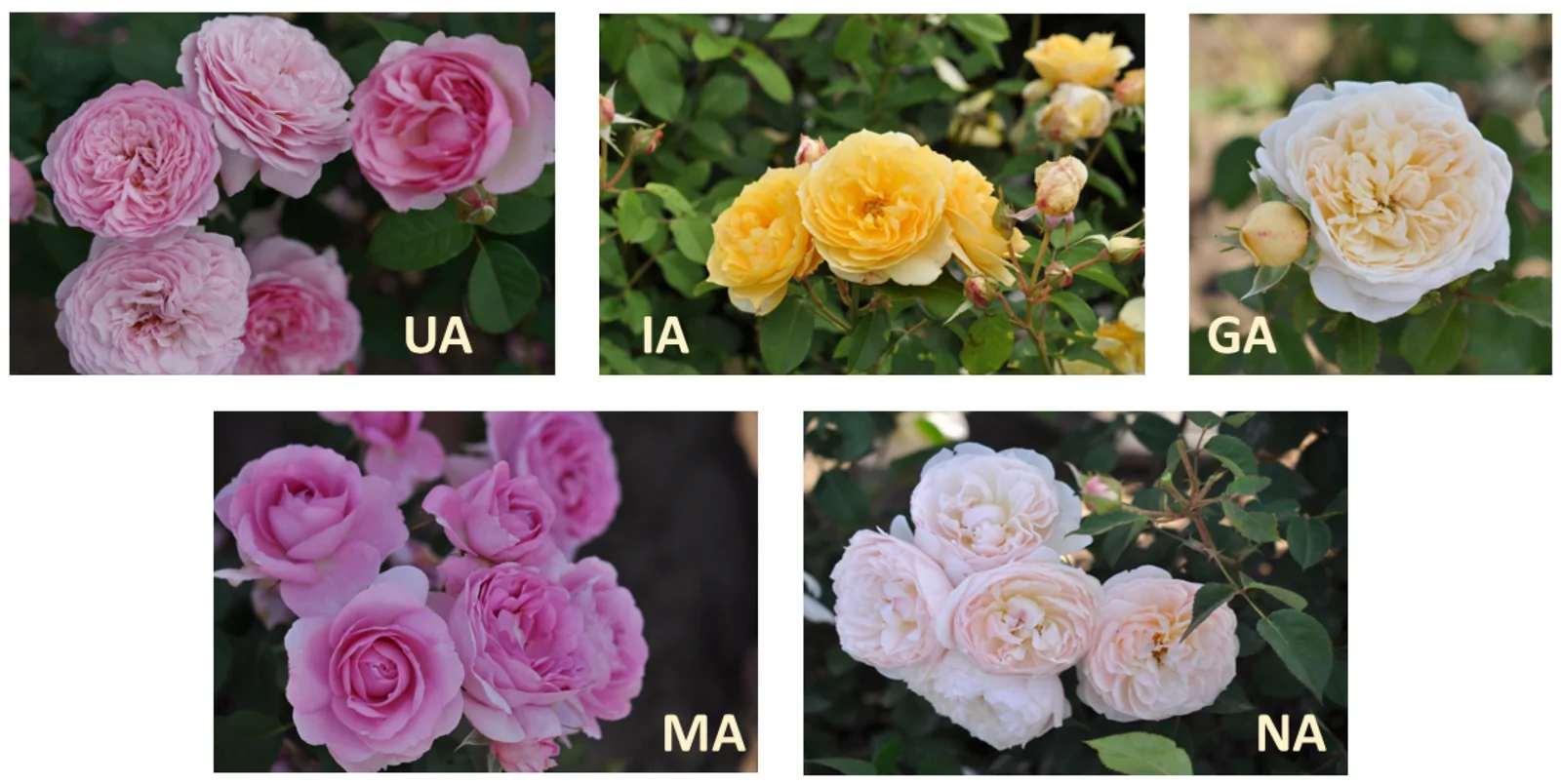
Rose genotypes investigated in this study: UA—‘Unique Aroma’, IA—‘Intense Aroma’, GA—‘Gentle Aroma’, MA—‘Magic Aroma’ and NA—‘Natural Aroma’.

The plant material (petals) used in the experiments consisted of five tetraploid (4n) edible garden rose cultivars—‘Unique Aroma’ (UA), ‘Intense Aroma’ (IA), ‘Gentle Aroma’ (GA), ‘Magic Aroma’ (MA) and ‘Natural Aroma’ (NA), shown in Figure 3. These cultivars are available on the market in Serbia, Italy, Hungary, France, Poland, Germany, the UK and the Netherlands. All garden rose genotypes belong to the *Rosa* × *hybrida* species and were cultivated using conventional breeding practices, with no use of chemical protection. From the outdoor experimental field of the private company ‘Pheno Geno Roses’ in Temerin, northern Serbia (coordinates: 45°24′19″ N 19°53′13″ E/45.105166° N 19.886833° E) 40 flowers from each cultivar were collected in late August 2021. Sensory analysis was performed immediately on fresh flowers, while the remaining samples were preserved at −80 °C until analysis.

### 3.2. Sensory Analysis

Fragrance evaluation as a qualitative trait was performed on freshly harvested flowers immediately after collection, by a panel of 45 volunteers (different genders, seniority, specialties and interests in roses), using the methodology described by Simin et al. [9].

### 3.3. Preparation of Methanol Extracts

Flowers were collected from multiple individual plants of each genotype. The petals were manually separated and pooled to obtain a composite sample representative of each genotype. An aliquot of the homogenized pooled petal material was then used for extraction and subsequent chemical analyses. Extraction was performed by maceration with 80% aqueous methanol at a solid-to-solvent ratio of 1:10 (*w*/*v*) for 48 h at room temperature without shaking. The obtained macerate was filtered and the extraction procedure was repeated once under the same conditions. Subsequently, the two filtrates were combined, evaporated to dryness in vacuo at 35 °C, after which the resulting dry extract was dissolved in dimethyl sulfoxide (DMSO) to a final concentration of 200 mg/mL and stored at −20 °C until analysis. All analyses were performed in triplicate.

### 3.4. Chemical Characterization of the Extracts

Total phenolic content (TPC) was determined using the Folin–Ciocalteu method, as previously described by Simin et al. [9]. Briefly, 30 μL of extract (0.1; 0.2 and 0.4 mg/mL) or gallic acid (standard, 0.625–80.0 μg/mL) was mixed with 150 μL 0.1 M Folin–Ciocalteu (FC) reagent and after incubation in the dark during 10 min, sodium carbonate was added (0.7 M, 120 μL). The color was developed for 2 h in the dark, after which the absorbance was measured at 720 nm. In the control, FC reagent was substituted with water. Results were expressed as milligrams of gallic acid equivalents per gram of dry extract (mg GAE/g de) and per gram of fresh weight (mg GAE/g fw).

Total flavonoid content (TFC) was determined by aluminium chloride assay as described by Simin et al. [9]. Briefly, 30 μL of petal extract (0.1; 0.2 and 0.4 mg/mL) or quercetin (0.625–80.0 μg/mL) was mixed with aluminium chloride (10%, 6 μL), sodium acetate (6 μL 1M), methanol (90 μL) and water (170 μL). The mixture was incubated for 30 min, and absorbance was measured at 415 nm. Results were expressed as milligrams of quercetin equivalents per gram of dry extract (mg QE/g de) and per gram of fresh weight (mg QE/g fw).

Total monomeric anthocyanin content (TAC) was determined using the pH differential method as described by Simin et al. [9]. The extract (15 μL) was mixed with 285 μL of acetate buffer pH 4 (sodium acetate 0.4 M and pH adjusted with acetic acid) or potassium chloride (0.025 M, pH adjusted to 1 with HCl). The mixture was incubated for 40 min and absorbance was measured at 520 nm and 700 nm. Results were expressed as micrograms of cyanidin 3-*O*-glucoside equivalents per gram of dry extract (µg CE/g de) and per gram of fresh weight (µg CE/g fw).

Quantitative analysis of 45 selected compounds was performed according to the method described by Orčić et al. [53]. Analysis was performed using Agilent Technologies 1200 Series high-performance liquid chromatograph (Agilent Technologies, Santa Clara, CA, USA) coupled with Agilent Technologies 6410A Triple Quad tandem mass spectrometer (Agilent Technologies, Santa Clara, CA, USA) with electrospray ion source and controlled by Agilent Technologies MassHunter Workstation software—Data Acquisition (ver. B04.00). Extracts were diluted to 20 mg/mL and 0.1 mg/mL with 50% aq MeOH. The sample (5 μL) was injected and compounds were separated on a Zorbax Eclipse XDB-C18 rapid resolution column (50 mm × 4.6 mm, 1.8 μm; Agilent Technologies, Santa Clara, CA, USA). The mobile phase consisted of A: 0.05% *v*/*v* aq HCOOH and B: MeOH with following gradient: 0 min 30% B, 6 min 70% B, 9 min 100% B, 12 min 100% B, with 3 min re-equilibration time (total analysis time per run 15 min) and a flow of 1 mL/min. Data were acquired in dynamic Multiple Reaction Monitoring (MRM) mode. Peak areas were determined using Agilent MassHunter Workstation Software—Qualitative Analysis (ver. B06.00). Calibration curves were plotted using OriginLab OriginPro (ver. 2019b) and used to calculate compound concentrations in the examined extracts. Results were expressed as micrograms of compound per gram of dry extract (µg/g de).

Vitamin C content was determined using a spectrophotometric method adapted for a 96-well plate as described by Simin et al. [9]. Briefly, rose petal extract (30 μL, 50 mg/mL) or ascorbic acid (5–320 μL/mL) was mixed with DCIP (270 μL 72 mg/L), and absorbance was measured at 515 nm after incubation of 5 min. Results were expressed as micrograms of vitamin C per gram of fresh weight (µg/g fw).

### 3.5. Antioxidant Activity

2,2-diphenyl-1-picrylhydrazyl (DPPH) and Ferric Reducing Antioxidant Power (FRAP) assays were used to evaluate the antioxidant capacity of rose petal extracts. Detailed methodology is described by Simin et al. [9]. For the DPPH assay, 10 μL of extract (0.047–3.0 mg/mL) was mixed with DPPH (100 μL 67.5 μM) and methanol (190 μL). The mixture was incubated for 1 h in the dark and absorbance was measured at 515 nm. The DPPH assay results were expressed as the EC_50_ value (the lowest concentration that achieves 50% radical neutralization, expressed as µg/mL). For FRAP assay, 10 μL of rose petal extract (0.1, 0.2 and 0.4 mg/mL) or ascorbic acid (1.0–160.0 μg/mL) was mixed with 290 μL of FRAP reagent (obtained by mixing 10 mM 2,4,6-tripyridyl-s-triazine (TPTZ) in 40 mM HCl, 0.02 M FeCl_3_ and acetate buffer 0.02 M pH 3.6, in a ratio of 1:1:10, respectively). The mixture was incubated for 6 min, and absorbance was measured at 593 nm. The results of the FRAP assay were expressed as milligrams of ascorbic acid equivalents per gram of dry weight (mg AAE/g de) and per gram of fresh weight (mg AAE/g fw).

### 3.6. Neuroprotective Activity

Neuroprotective activity was evaluated by the ability of extracts to inhibit the activity of acetylcholinesterase (AChE). For this, the method described by Pintać et al. was utilized [54]. Briefly, AChE (0.5 U/mL, 20 μL) was mixed with 20 mM TRIS-HCl buffer (110 μL, pH 8) and 10 μL of petal extract (1 mg/mL) or eserine (1.35 × 10^−6^–1.38 × 10^−3^ mg/mL). In the blank probe, AChE was substituted with 20 mM TRIS-HCl buffer pH 7.5, while in the control, instead of the sample, 20 mM TRIS-HCl buffer pH 8 was added. After incubation of 15 min at 37 °C with constant shaking, 40 μL of 3 mM 5,5′-dithio-bis-(2-nitrobenzoic acid) solution and 20 μL of 15 mM acetylcholine iodide were added. The absorbance was measured at 412 nm after 3 min of the reaction start. Results were expressed as percent of inhibition and as nanograms of eserine equivalents per gram of dry extract (ng EE/g de).

### 3.7. Hypoglycemic Activity

The hypoglycemic activity was evaluated by ability of extracts to inhibit the activity of enzymes α-amylase and α-glucosidase, as described by Simin et al. [8]. For α-amylase, 90 μL of α-amylase solution (0.1 μg/mL in 20 mM phosphate buffer pH 6.9), was mixed with 10 μL of extracts (5.0 mg/mL) or acarbose (0.125–3.0 mg/mL), after which, 80 μL of 0.05% starch (dissolved in 20 mM phosphate buffer pH 6.9) was added and incubated at 37 °C with constant shaking for 10 min. The reaction was stopped by adding cold HCl (100 μL 1 M), followed by adding 20 μL of Lugol solution, and absorbance was measured at 620 nm. Results of α-amylase inhibition were expressed as percent of inhibition and as milligrams of acarbose equivalents per gram of dry extract (mg ACAE/g de). For α-glucosidase, phosphate buffer (100 μL 0.1M pH 6.8) was mixed with 10 μL of α-glucosidase (0.1 U/mL in 0.1 M phosphate buffer pH 6.8), 20 μL of extract (20 μg/mL) or acarbose (0.31–10.0 mg/mL) and 20 μL of substrate (5 mM *p*-nitrophenyl α-D-glucoside in 0.1 M phosphate buffer pH 6.8). The mixture was incubated at 37 °C with constant shaking for 15 min. After incubation, 80 μL of 0.2 M Na_2_CO_3_ was added and absorbance was measured at 400 nm. Results of α-glucosidase inhibition were expressed as percent of inhibition and gram of acarbose equivalents per gram of dry extract (g ACAE/g de).

## 4. Conclusions

Edible rose petals are emerging not only as a culinary trend but also as promising sources of bioactive nutrients with notable functional potential. Their increasing incorporation, not only in Michelin-star gastronomy but also in traditional and luxury cuisines globally, highlights their growing culinary relevance and emphasizes the need for rigorous scientific validation. As chefs and consumers alike seek natural, visually appealing and health-promoting ingredients, there is a corresponding rise in demand for evidence-based research into the nutritional composition, safety and functional properties of edible flowers.

Based on this study’s findings, flowers from novel genotypes of *R. hybrida* cultivated in Serbia should be appreciated not only for their esthetic and aromatic qualities but also as rich sources of vitamin C and polyphenolic compounds with numerous health-promoting effects. The substantial phenolic content and confirmed biological activities underscore their potential as valuable sources of bioactive constituents with diverse applications in the food and nutraceutical industries. The MA cultivar, which exhibited the highest contents of TPC, TAC, vitamin C, rutin, quercitrin, and quercetin 3-*O*-glucoside+galactoside, as well as the highest activity in all assays (DPPH, FRAP, and enzyme inhibition), may be particularly interesting for future studies. Moreover, this cultivar is characterized by a distinctive fragrance profile, with top notes of sour apple, camphor, and anise; middle notes of sweet honey, spicy and peppery aromas; and base notes of pine wood, earthy, musky, and balsamic aromas. These sensory characteristics, together with its high content of bioactive compounds and antioxidant and enzyme-inhibitory activities, suggest that this cultivar may also have potential for use as an aromatic ingredient in culinary applications.

## Figures and Tables

**Table 1:** Qualitative and quantitative generative characteristics of the investigated rose genotypes.

Genotype/Trait	IA	GA	NA	MA	UA
Flowering shoot					
Flowering laterals	Absent	Present	Absent	Absent	Absent
Number of flowering shoots	1.8 ± 0.84 ^b^ *	0 ^c^	3.4 ± 1.14 ^b^	2.6 ± 0.55 ^b^	4.4 ± 1.14 ^a^
Number of flowering laterals	0 ^b^	2.6 ± 0.54 ^a^	0 ^b^	0 ^b^	0 ^b^
Number of flowers per lateral	0 ^b^	3.2 ± 1.30 ^a^	0 ^b^	0 ^b^	0 ^b^
Flower					
Type	Double	Double	Double	Double	Double
Color group	Yellow	Pale yellow	Pale yellow	Pale pink	Pale pink
Color of center (type double)	Yellow	Yellow	Pale orange	Pink	Pink
Shape	Irregularly rounded	Rounded	Rounded	Rounded	Irregularly rounded
Profile of upper part	Flat	Flat	Flat	Flat	Flat
Profile of lower part	Concave	Concave	Convex	Concave	Concave
Fragrance (1–5 scale)	5	4	4	5	5
Number of petals	91.6 ± 4.39 ^b^	148.4 ± 19.9 ^a^	73.2 ± 8.87 ^bc^	52 ± 5.24 ^c^	148 ± 20.8 ^a^
Flower diameter (cm)	8.04 ± 0.49 ^a^	6.88 ± 0.82 ^ab^	7.4 ± 0.43 ^ab^	6.72 ± 0.83 ^ab^	6.66 ± 0.88 ^b^
Petal length (cm)	4.66 ± 0.36 ^a^	3.60 ± 0.39 ^b^	4.42 ± 0.25 ^ab^	4.8 ± 0.57 ^a^	3.78 ± 0.56 ^b^
Petal width (cm)	3.48 ± 0.33 ^ab^	3.36 ± 0.49 ^ab^	4.04 ± 0.62 ^a^	4.18 ± 0.61 ^a^	2.84 ± 0.42 ^b^

* Letters a–c denote statistically significant difference between genotypes based on Tukey’s Honest Significant Difference test (*p* ≤ 0.05). Genotypes abbreviations: IA—‘Intense Aroma’, GA—‘Gentle Aroma’, NA—‘Natural Aroma’, MA—‘Magic Aroma’, UA—‘Unique Aroma’.

**Table 2:** Panelist assessments of fragrance notes: top, middle and base.

Fragrance	IA	GA	NA	MA	UA
Top notes					
Orange	6.98	8.64	0.81	0.90	3.17
Lemon	33.33	20.99	2.44	6.31	17.46
Sour apple	12.40	12.35	13.01	17.12	11.11
Mandarin	6.98	11.11	0.81	6.31	4.76
Bergamot	1.55	6.17	10.57	9.01	2.38
Grapefruit	6.20	3.70	5.69	0.90	12.70
Camphor	4.65	3.70	14.63	14.41	19.05
Mint	11.63	9.88	12.20	17.12	3.17
Anise	5.43	6.17	26.02	18.02	11.11
Eucalyptus	9.30	9.88	4.07	0.00	10.32
Sweet fennel	1.55	7.41	9.76	9.91	4.76
Middle notes					
Flowery	12.96	12.43	4.90	7.95	10.49
Rose-like	7.29	9.73	3.67	5.30	5.24
Jasmine	2.02	8.11	4.90	7.20	5.62
Elderflower	6.07	5.41	9.80	5.30	3.37
Lilac	1.62	2.16	1.63	3.41	5.99
Forest	10.12	4.32	8.57	5.30	9.36
Herbaceous	6.88	7.03	4.90	4.17	4.87
Grass	3.24	6.49	5.71	6.06	6.37
Fruity	15.76	8.11	3.67	6.82	11.61
Sweet honey	6.48	12.97	11.84	12.50	5.62
Raspberry	0.40	2.16	0.82	2.27	3.37
Peach	3.64	3.78	1.22	1.52	0.75
Pear	5.26	2.70	2.45	0.76	2.62
Strawberry	0.40	1.08	1.63	0.38	1.50
Spicy	3.24	3.24	13.06	9.85	5.62
Peppery	0.81	0.54	4.08	7.95	3.00
Nutty	3.24	3.24	3.67	2.65	3.00
Hot	6.88	1.08	5.71	3.41	3.37
Cinnamon	1.21	2.16	0.82	1.14	2.25
Cloves	1.21	1.08	2.45	2.65	2.62
Nutmeg	1.21	2.16	4.49	3.41	3.37
Base notes					
Pine wood	6.19	3.45	3.68	10.48	6.96
Moss	12.37	10.34	5.15	4.84	3.48
Woody	2.06	3.45	7.35	5.65	8.70
Resin	7.22	8.05	10.29	6.45	11.30
Earthy	13.40	2.30	12.50	15.32	13.04
Sandalwood	5.15	19.54	13.24	7.26	10.43
Balsamic	12.37	10.34	13.97	15.32	11.30
Tobacco-like	0.00	1.15	5.15	7.26	3.48
Musky	17.53	17.24	13.97	16.94	13.04
Myrtle	7.22	6.90	8.09	4.03	8.70
Vanilla	16.49	17.24	6.62	6.45	9.57

IA—‘Intense Aroma’, GA—‘Gentle Aroma’, NA—‘Natural Aroma’, MA—‘Magic Aroma’, UA—‘Unique Aroma’.

**Table 3:** Panel scoring for general impression by the present fragrance components.

Emotional Response	IA	GA	NA	MA	UA
Bright	14.29	9.48	2.05	0.00	4.20
Mild	8.40	17.24	1.37	0.78	10.08
Light	11.76	8.62	0.00	4.65	9.24
Cold	2.52	0.86	3.42	2.33	6.72
Fresh	13.45	12.07	2.74	5.43	8.40
Rich	9.24	9.48	8.22	8.53	6.72
Intensive	6.72	0.86	16.44	23.26	10.92
Warm	7.56	5.17	4.79	3.10	5.04
Wet	4.20	4.31	3.42	3.88	5.04
Dry	0.84	1.72	2.74	3.88	0.84
Heavy	2.52	2.59	19.86	14.73	5.88
Powerful	5.88	0.86	12.33	5.43	5.88
Inspiring	5.88	10.34	8.22	6.20	9.24
Irritating	5.04	0.00	14.38	15.50	10.08
Seductive	0.84	10.34	0.00	1.55	0.00
Calming	0.84	6.03	0.00	0.78	1.68

IA—‘Intense Aroma’, GA—‘Gentle Aroma’, NA—‘Natural Aroma’, MA—‘Magic Aroma’, UA—‘Unique Aroma’.

**Table 4:** Total phenolic, flavonoid, anthocyanin contents and content of vitamin C in rose petal extracts.

	MA	IA	NA	UA	GA
TPC					
(mg GAE/g de)	240 ± 3.71 ^a^ *	124 ± 11.5 ^cd^	180 ± 5.48 ^b^	142 ± 1.67 ^c^	122 ± 8.41 ^d^
(mg GAE/g fw)	23.4 ± 0.36 ^a^	10.9 ± 1.01 ^c^	15.2 ± 0.46 ^b^	14.5 ± 0.17 ^b^	12.1 ± 0.83 ^c^
TFC					
(mg QE/g de)	27.3 ± 2.11 ^c^	19.9 ± 1.48 ^d^	42.7 ± 1.33 ^a^	18.1 ± 1.37 ^d^	34.9 ± 2.89 ^b^
(mg QE/g fw)	2.67 ± 0.21 ^b^	1.74 ± 0.13 ^c^	3.59 ± 0.11 ^a^	1.85 ± 0.14 ^c^	3.46 ± 0.29 ^a^
TAC					
(µg CE/g de)	1749 ± 57.3 ^a^	<200	<200	270 ± 14.7 ^b^	<200
(µg CE/g fw)	171 ± 5.60 ^a^	<17.5	<16.8	27.7 ± 1.44 ^b^	<19.8
Vitamin C					
(µg/g fw)	309 ± 12.1 ^b^	236 ± 0.99 ^c^	244 ± 11.9 ^bc^	254 ± 1.89 ^bc^	258 ± 7.82 ^b^

* Letters a–d denote statistically significant difference between genotypes based on one-factor ANOVA followed by Tukey-HSD test (*p* ≤ 0.05); MA—‘Magic Aroma’, IA—‘Intense Aroma’, NA—‘Natural Aroma’, UA—‘Unique Aroma’, GA—‘Gentle Aroma’, TPC—Total phenolic content, GAE—Gallic acid equivalents, TFC—Total flavonoid content, QE—Quercetin equivalents, TAC—Total monomeric anthocyanins content, CE—Cyanidin-3-*O*-glucoside equivalents.

**Table 5:** The content (µg/g de) of selected compounds in rose petal extracts determined by LC-MS/MS.

	MA	IA	NA	UA	GA
Organic acids					
Quinic acid	14,797 ± 1480 ^a^ *	**13,163 ± 1316 ^a^**	**16,366 ± 1637 ^a^**	**9386 ± 939 ^b^**	**14,281 ± 1428 ^a^**
Hydroxybenzoic acids					
Protocatechuic acid	12.2 ± 0.98 ^a^	1.73 ± 0.14 ^c^	1.06 ± 0.08 ^c^	7.83 ± 0.63 ^b^	0.95 ± 0.08 ^c^
Gallic acid	41.8 ± 3.76 ^a^	32.8 ± 2.95 ^b^	17.7 ± 1.60 ^c^	35.8 ± 3.22 ^ab^	17.7 ± 1.59 ^c^
Hydroxycinnamic acids					
*p*-Coumaric acid	4.04 ± 0.36 ^b^	1.19 ± 0.11 ^c^	6.13 ± 0.55 ^a^	0.44 ± 0.04 ^d^	0.29 ± 0.03 ^d^
Caffeic acid	<0.60 **	0.88 ± 0.06	<0.60	<0.60	<0.60
Ferulic acid	nd ***	<0.60	nd	nd	0.73 ± 0.07
Chlorogenic acid	1.41 ± 0.07 ^d^	7.75 ± 0.39 ^b^	0.49 ± 0.02 ^e^	5.80 ± 0.29 ^c^	11.6 ± 0.58 ^a^
Flavanols					
Catechin	22.9 ± 2.29 ^d^	219 ± 21.9 ^a^	53.2 ± 5.32 ^cd^	72.3 ± 7.23 ^c^	105 ± 10.5 ^b^
Epicatechin	nd	nd	<1.20	<1.20	nd
Epicatechin gallate	<19.6	nd	<19.6	<19.6	<19.6
Flavanones					
Naringenin	0.35 ± 0.02 ^c^	1.09 ± 0.08 ^a^	1.13 ± 0.08 ^a^	0.70 ± 0.05 ^b^	0.39 ± 0.03 ^c^
Flavones					
Baicalein	nd	nd	nd	<1.20	<1.20
Luteolin	0.39 ± 0.02 ^a^	0.44 ± 0.02 ^a^	<0.30	0.41 ± 0.02 ^a^	0.41 ± 0.02 ^a^
Luteolin 7-*O*-glucoside	nd	1.01 ± 0.03	nd	nd	nd
Apigenin	nd	nd	nd	0.11 ± 0.01	nd
Apigenin 7-*O*-glucoside	<0.08	0.10 ± 0.01 ^c^	0.24 ± 0.01 ^a^	0.15 ± 0.01 ^b^	0.14 ± 0.01 ^b^
Vitexin	0.30 ± 0.02 ^b^	0.10 ± 0.01 ^c^	0.39 ± 0.02 ^a^	<0.08	nd
Chrysoeriol	0.21 ± 0.006 ^b^	0.52 ± 0.016 ^a^	<0.08	0.16 ± 0.005 ^c^	0.13 ± 0.004 ^d^
Flavonols					
Quercetin	24.5 ± 7.34 ^a^	14.1 ± 4.22 ^ab^	11.9 ± 3.56 ^b^	11.7 ± 3.51 ^b^	6.25 ± 1.88 ^b^
Rutin	**1506 ± 45.2 ^a^**	598 ± 17.9 ^c^	251 ± 7.53 ^d^	616 ± 18.5 ^c^	954 ± 28.6 ^b^
Quercetin 3-*O*-glucoside+galactoside	**10,993 ± 660 ^a^**	**4630 ± 278 ^bc^**	**4231 ± 254 ^c^**	**5284 ± 317 ^b^**	**1624 ± 97.5 ^d^**
Quercitrin	**7663 ± 460 ^a^**	**1007 ± 60.4 ^c^**	**2697 ± 162 ^b^**	**2294 ± 138 ^b^**	538 ± 32.3 ^c^
Kaempferol	3.51 ± 0.25 ^c^	43.4 ± 3.04 ^a^	22.1 ± 1.55 ^b^	21.0 ± 1.47 ^b^	18.0 ± 1.26 ^b^
Kaempferol 3-*O*-glucoside	**5760 ± 230 ^d^**	**38,655 ± 1546 ^a^**	**40,658 ± 1626 ^a^**	**30,689 ± 1228 ^b^**	**24,468 ± 979 ^c^**
Isorhamnetin	<0.15	<0.15	nd	nd	nd
Chemically quantified extract amount **** (mg/g de)	40.8	58.4	64.3	48.4	42.0

* Letters a–e denote statistically significant difference between genotypes based on one-factor ANOVA followed by Tukey-HSD test (*p* ≤ 0.05); ** below the limit of quantification (LOQ); *** not detected; **** sum of the contents of all detected compounds using LC-MS/MS; the values higher than 1000 are marked with bold letters; MA—‘Magic Aroma’ IA—‘Intense Aroma’, NA—‘Natural Aroma’, UA—‘Unique Aroma’, GA—‘Gentle Aroma’.

**Table 6:** Antioxidant, neuroprotective and hypoglycemic potential of rose petal extracts.

	MA	IA	NA	UA	GA
DPPH					
EC_50_ (µg/mL)	18.4 ± 0.80 ^c^ *	44.9 ± 3.06 ^a^	26.8 ± 1.47 ^b^	43.1 ± 3.04 ^a^	22.8 ± 1.44 ^bc^
FRAP					
(mg AAE/g de)	173 ± 15.0 ^a^	62.6 ± 2.73 ^c^	112 ± 7.69 ^b^	79.1 ± 5.03 ^c^	117 ± 4.63 ^b^
(mg AAE/g fw)	16.9 ± 1.47 ^a^	5.47 ± 0.24 ^d^	9.44 ± 0.65 ^c^	8.10 ± 0.52 ^c^	11.6 ± 0.46 ^b^
AChE					
(ng EE/g de)	12.9 ± 1.45 ^a^	9.33 ± 0.89 ^b^	10.9 ± 0.41 ^ab^	7.14 ± 0.57 ^bc^	6.33 ± 0.52 ^c^
(%)	33.3 ± 2.78 ^a^	27.8 ± 1.80 ^b^	30.8 ± 0.72 ^ab^	22.9 ± 1.40 ^c^	20.9 ± 1.99 ^c^
α-Amylase					
(mg ACAE/g de)	46.2 ± 5.26 ^a^	30.8 ± 5.22 ^b^	57.9 ± 4.07 ^a^	33.0 ± 4.35 ^b^	N/A
(%)	5.85 ± 0.63 ^a^	3.75 ± 0.62 ^b^	6.97 ± 0.48 ^a^	4.01 ± 0.52 ^b^	N/A
α-Glucosidase					
(g ACAE/g de)	212 ± 4.00 ^a^	12.2 ± 0.48 ^e^	128 ± 0.08 ^b^	33.1 ± 0.39 ^d^	116 ± 8.39 ^c^
(%)	81.6 ± 1.56 ^a^	23.7 ± 0.67 ^d^	74.9 ± 3.78 ^ab^	46.1 ± 3.77 ^c^	70.8 ± 1.38 ^b^

* Letters a–e denote statistically significant difference between genotypes based on one-factor ANOVA followed by Tukey-HSD test (*p* ≤ 0.05); MA—‘Magic Aroma’, IA—‘Intense Aroma’, NA—‘Natural Aroma’, UA—‘Unique Aroma’, GA—‘Gentle Aroma’, IC_50_—extract concentration that neutralizes 50% of DPPH radicals, AAE—ascorbic acid equivalents, de—dry extract, fw—fresh weight, EE—eserine equivalents, ACAE—acarbose equivalents.

## Data Availability

The original contributions presented in this study are included in the article. Further inquiries can be directed to the corresponding author.

## Abbreviations

The following abbreviations are used in this manuscript:

CAGR	compound annual growth rate
GA	‘Gentle Aroma’
MA	‘Magic Aroma’
PL	petal length
PW	petal width
IA	‘Intense Aroma’
NA	‘Natural Aroma’
UA	‘Unique Aroma’
FL	flowering laterals
NFS	number of flowering shoots
NFL	number of flowering laterals
NF/L	number of flowers per lateral
TP	type
CG	color group
CC	color of center
SH	shape
PUP	profile of upper part
PLP	profile of lower part
FG	fragrance
NOP	number of petals
DM	flower diameter
LC-MS/MS	liquid chromatography coupled with tandem mass spectrometry
TPC	total phenolic content
GAE	gallic acid equivalents
de	dry extract
fw	fresh weight
TFC	total flavonoid content
QE	quercetin equivalents
TAC	total monomeric anthocyanins content
CE	cyanidin-3-*O*-glucoside equivalents
nd	not detected
LOQ	limit of quantification
PCA	principal component analysis
PC1	Principal Component 1
PC2	Principal Component 2
DPPH	2,2-diphenyl-1-picrylhydrazyl
IC_50_	extract concentration that neutralizes 50% of DPPH radicals
FRAP	Ferric Reducing Antioxidant Power assay
AAE	ascorbic acid equivalents
AChE	acetylcholinesterase
EE	eserine equivalents
AKAE	acarbose equivalents
Nrf2	nuclear factor erythroid 2-related factor 2
